# Risk Factors Associated with Adverse Events Leading to Methotrexate Withdrawal in Elderly Rheumatoid Arthritis Patients: A Retrospective Cohort Study

**DOI:** 10.3390/jcm13071863

**Published:** 2024-03-24

**Authors:** Felipe Alexis Avalos-Salgado, Laura Gonzalez-Lopez, Sergio Gonzalez-Vazquez, Juan Manuel Ponce-Guarneros, Aline Priscilla Santiago-Garcia, Edna Lizeth Amaya-Cabrera, Reynaldo Arellano-Cervantes, J. Ahuixotl Gutiérrez-Aceves, Miriam Fabiola Alcaraz-Lopez, Cesar Arturo Nava-Valdivia, Fabiola Gonzalez-Ponce, Norma Alejandra Rodriguez-Jimenez, Miguel Angel Macias-Islas, Edgar Ricardo Valdivia-Tangarife, Ana Miriam Saldaña-Cruz, Ernesto German Cardona-Muñoz, Jorge Ivan Gamez-Nava

**Affiliations:** 1 Programa de Doctorado en Farmacología, Centro Universitario de Ciencias de la Salud, Universidad de Guadalajara, Guadalajara 44340, Mexico; felipe.asalgado@alumnos.udg.mx (F.A.A.-S.); ahuixotl.gutierrez@alumnos.udg.mx (J.A.G.-A.); 2 Research Group for Factors Related to Therapeutic Outcomes in Autoimmune Diseases, Centro Universitario de Ciencias de la Salud, Universidad de Guadalajara, Guadalajara 44340, Mexicofabiola.gonzalez@academicos.udg.mx (F.G.-P.); norma.rodriguezj@academicos.udg.mx (N.A.R.-J.); german.cardona@academicos.udg.mx (E.G.C.-M.); 3 Programa de Maestria Salud Publica, Departamento de Salud Pública, Centro Universitario de Ciencias de la Salud, Universidad de Guadalajara, Guadalajara 44340, Mexico; 4 Instituto de Terapéutica Experimental y Clínica, Departamento de Fisiología, Centro Universitario de Ciencias de la Salud, Universidad de Guadalajara, Guadalajara 44340, Mexico; 5 Hospital General Regional 110, Instituto Mexicano del Seguro Social, Guadalajara 44716, Mexico; 6 Instituto Mexicano del Seguro Social, Unidad de Medicina Familiar No. 97, Magdalena 46474, Mexico; 7 Departamento de Reumatología, Hospital Civil Fray Antonio Alcalde, Guadalajara 45019, Mexico; edna.amaya.cabrera@gmail.com; 8 Departamento de Ciencias del Movimiento Humano, Centro Universitario de Ciencias de la Salud, Universidad de Guadalajara, Guadalajara 44340, Mexico; 9 Departamento de Medicina Interna-Reumatología, Hospital General Regional Núm. 46, Instituto Mexicano del Seguro Social, Guadalajara 44910, Mexico; 10 Departamento de Microbiología y Patología, Centro Universitario de Ciencias de la Salud, Universidad de Guadalajara, Guadalajara 44340, Mexico; 11 Departamento de Neurociencias, Centro Universitario de Ciencias de la Salud, Universidad de Guadalajara, Guadalajara 44340, Mexico; miguelangelmacias@hotmail.com (M.A.M.-I.);

**Keywords:** rheumatoid arthritis in elderly, methotrexate, adverse events, withdrawals, treatment persistence, retrospective cohort studies

## Abstract

**Background:** Rheumatoid arthritis (RA) in elderly population represents a challenge for physicians in terms of therapeutic management. Methotrexate (MTX) is the first-line treatment among conventional synthetic-disease-modifying anti-rheumatic drugs (cs-DMARDs); however, it is often associated with adverse events (AEs). Therefore, the objective of this study was to identify the incidence and risk factors of MTX discontinuation due to AEs in elderly patients with RA in a long-term retrospective cohort study. **Methods:** Clinical sheets from elderly RA patients taking MTX from an outpatient rheumatology consult in a university centre were reviewed. To assess MTX persistence, we used Kaplan–Meir curves and Cox regression models to identify the risk of withdrawing MTX due to adverse events. **Results:** In total, 198 elderly RA patients who reported using MTX were included. Of them, the rates of definitive suspension of MTX due to AEs were 23.0% at 5 years, 35.6% at 10 years and 51.7% at 15 years. The main organs and system involved were gastrointestinal (15.7%) and mucocutaneous (3.0%). Factors associated with withdrawing MTX due to AEs were MTX dose ≥ 15 mg/wk (adjusted HR: 2.46, 95% CI: 1.22–4.96, *p =* 0.012); instead, the folic acid supplementation was protective for withdrawal (adjusted HR: 0.28, 95% CI: 0.16–0.49, *p <* 0.001). **Conclusions:** Higher doses of MTX increase the risk of withdrawals in elderly RA, while folic acid supplementation reduces the risk. Therefore, physicians working in therapeutic management for elderly patients using MTX must focus on using lower MTX doses together with the concomitant prescription of folic acid.

## 1. Introduction

Rheumatoid arthritis (RA) is a systemic, autoimmune inflammatory disease that affects synovial joints, causing chronic pain, bone erosions and disability [1]. The RA prevalence ranges from 0.5 to 1% worldwide [2]. Early onset of pharmacological treatment with conventional synthetic disease-modifying anti-rheumatic drugs (cs-DMARDs), constitutes the corner stone of the therapy directed to control the disease activity and decrease the progression of the disease. Methotrexate (MTX) is the synthetic DMARD prescribed as the first line of treatment [3,4]. MTX is a folic acid analog which exerts its therapeutic effects by inhibiting dihydrofolate reductase (DHFR), leading to decreased levels of tetrahydrofolate (THF), a vital component in purine synthesis. This depletion of purine nucleotides, including aminoimidazole carboxamide ribonucleotide transformylase (AICART), disrupts the synthesis of purines, crucial for cell proliferation. Consequently, methotrexate impedes the hyperactive immune response characteristic of RA, reducing inflammation and joint damage [5]. In many countries, including Mexico, MTX is the cs-DMARD most often prescribed to treat RA in around 70% of the patients [6]. However, most of the patients using MTX correspond to persons between the 4th and 5th decade of life and the information derived from using this drug in elderly patients is still limited, while in non-elderly RA patients, there is a wide variability in the time of using MTX [7,8,9,10,11]. The lack of persistence is one of the main factors that limits the effectiveness of MTX, and frequently, the withdrawals of the drug are caused by adverse events (AEs), which, depending on their severity, can lead to treatment discontinuation, from causing distress for the patient (mild) to more serious health complications (severe) [12]. Various factors associated with AEs that lead to MTX discontinuation have been investigated: increased body mass index (BMI) [13], higher pain perception [13], increased ALT levels [13], MTX dose [14], disease duration [14], lack of folate supplementation [14], age [14,15], sex [15], etc.

The World Health Organization (WHO) estimates that between 2015 and 2050, the proportion of the global population aged 60 years and older will nearly double. [16]. The presence of RA in elderly individuals is an important challenge for rheumatologists, internal medicine specialists and primary-care physicians. Studies performed in outpatient rheumatology clinics indicate that at least 20% of the patients with RA are older than 60 years [17]. However, the literature regarding the behavior of MTX and other drugs in this population is scarce. To date, there are few studies that have assessed MTX withdrawal due to adverse events in the elderly RA population during long-term therapy. Therefore, the present study aimed to identify the incidence and risk factors for withdrawals of MTX due to adverse events in elderly patients with RA in a long-term retrospective cohort study.

## 2. Materials and Methods

### 2.1. Study Design and Clinical Setting

Study design: retrospective cohort study. Three trained researchers performed a systematic assessment of clinical charts from elderly patients with RA from a 1000-patient cohort study who attended an outpatient rheumatology consultation therapeutic university centre from 1 January 1980 to 31 December 2016. The referred center mostly accommodates patients from Jalisco state in Mexico. The study was performed from September 2021 to January 2023.

### 2.2. Inclusion, Exclusion and Elimination Criteria

We included patients with RA diagnosed by a rheumatologist meeting the American Rheumatism Association 1987 revised criteria for RA [18]. These patients were ≥60 years old at the time of the first consult with the rheumatologist, were prescribed oral MTX for treating the joint manifestations of RA and had more than one visit to the rheumatologist for clinical assessment of evolution and response to treatment.

Patients with only one clinical visit were excluded. Other exclusion criteria were if at the baseline patients had any of the following conditions: cancer, chronic kidney disease (stage 3B or higher), liver diseases, hepatic insufficiency, active infectious diseases, untreated immunodeficiency, interstitial pneumonitis, pulmonary fibrosis, recent vaccination or hypersensitivity, alongside patients with overlapping syndrome (presence of RA plus symptoms of systemic lupus erythematosus, systemic scleroderma, and polymyositis).

### 2.3. Ethics

This study was approved by the following committees: Ethics in Research committee (CEI-CUCS) and Committee of Research (CI-CUCS) at the University Centre of Health Sciences (CUCS), University of Guadalajara, approval code CI-04021. This research protocol followed the Ethical Principles for Medical Research Involving Human Subjects described in the Helsinki Declaration [19].

### 2.4. Study Development

Demographic and clinical data were ascertained by three trained researchers who reviewed elderly RA patients’ clinical charts who attended the public hospital. Information recollected was classified as:

(a) Sociodemographic variables: gender, age, type of insurance, body mass index (BMI), alcohol consumption, smoking;
(b) Comorbid diseases: hypertension, diabetes mellitus type 2, obesity, depression and other comorbid diseases;
(c) Disease characteristics: disease duration, articular and extraarticular manifestations, functional classification;
(d) Pharmacological treatment (cs-DMARDS use, Methotrexate, Sulfasalazine, Antimalarials, other cs-DMARDS), persistence of treatment (years), combined cs-DMARD therapy—use of 2 or more cs-DMARDs simultaneously—and any other drug prescribed, such as glucocorticoids, non-steroidal anti-inflammatory drugs, analgesics, painkillers, antiacids, or antihypertensives, alongside folic acid supplementation;
(e) Safety: adverse events that led to MTX discontinuation alongside MTX dose and usage time at the time of their appearance. Only adverse events that led patients to stop their MTX treatment for more than 90 days were counted. Additionally, these were classified based on the organ and system affected and the specific type of event.
(f) Adverse events were considered as reported by the rheumatologist in the clinical charts at the time of each visit. Because this is a retrospective cohort, we were unable to identify the adverse events using prespecified definitions; however, the guidelines/recommendations to identify adverse events associated with MTX in our institution are described briefly as follows:
  - Gastritis, gastropathies and gastrointestinal manifestations. These included gastric or duodenal mucosal injury, nausea, vomiting, mucosal ulcers, loss of appetite and epigastralgia.
  - Transaminitis (elevated transaminases): presence of alanine transaminase (ALT) and aspartate transaminase (AST), higher than upper limits of normal (ULN) cutoff values of the reference laboratory; the normal values in our laboratory were as follows: ALT: normal range 5–50 IU/L; AST: normal range 10 to 34 IU/L. Severe transaminitis was considered an increase of ALT or AST > 3-fold ULN in two consecutive visits.
  - Diagnosis of bleeding diverticulitis was performed by gastroenterologist based on symptoms of recurrent mild abdominal pain and distension plus rectal bleeding, corroborated with diverticula images in abdominal CT scan.
  - Oral ulcers: symptoms of painful and observation of well-defined small ulcers (yellow or white rounded by erythema) in mouth and throat plus diverse difficulty in swallowing food with presence of small mucosal erosions/ulcerations on oral mucosa and/or tongue. Chronologically associated with MTX use and disappearing after this DMARD withdrawn (corroborated by dermatologist).
  - Alopecia and hair loss: hair loss temporally correlated with the use of MTX, disappeared when MTX was withdrawn (corroborated by dermatologist).
  - Abnormal blood counts; definitions:
    ○ Anemia: hemoglobin level of <115 g/L;
    ○ Leukopenia: peripheral blood leukocyte count < 3.0 × 10^9^/L;
    ○ Neutropenia: neutrophil count of <1.8 × 10^9^/L;
    ○ Lymphopenia: lymphocytes count < 1.1 × 10^9^/L;
    ○ Thrombocytopenia: platelet count of <100 × 10^9^/L;
    ○ Thrombocytosis: platelet count of >450 × 10^9^/L.
  - Interstitial pneumopathy: persistent symptoms of dyspnea and dry cough, plus findings of scattered or diffuse and patchy, ground glass opacity with images of reticular involvement identified by high resolution computed tomography (HRCT) and corroborated by a pneumologist. Pulmonary fibrosis was diagnosed by findings of honeycombing images (clustered cystic airspaces) located in subpleural region, with well-defined walls and diameters >0.5 cm, observed in HRCT corroborated by radiologist and pneumologist.
  - Dermatosis attributable to MTX: cutaneous lesions of erythematous indurated papules located on proximal areas of the extremities with a direct chronologic correlation with MTX therapy corroborated by dermatologist. These lesions disappeared when MTX was withdrawn and had response to corticosteroids (topical or systemics).
  - Weakness as persistent symptoms of fatigue or tiredness.
  - Weight loss: >10% in kilograms, obtained from the difference between weight in the last visit (index visit)—weight in the previous visit.
  - Recurrent infections: corroborated by persistent positive cultures or other accepted method.
  - Urinary lithiasis: presence of stones observed in kidney or urinary tract using ultrasound or computed tomography (CT).

### 2.5. Statistical Analysis

The incidence rate for adverse events that led to MTX suspension was computed, and each adverse event was reported based on the organ and system affected along with their specific description. Independent Student’s *t*-tests were used for comparisons of quantitative variables between groups; chi-square tests (or Fischer exact tests if required) were used for comparisons of proportions between groups. The cumulative drug survival probability of MTX treatment persistence was analyzed using the Kaplan–Meier method, where the reasons for censoring were the MTX continuation at the end of the study or loss to follow-up, and plots were used to determine the incidence density of MTX discontinuation due to AEs. Univariate and multivariate Cox proportional hazards regression models were used to assess potential predictors for MTX discontinuation due to AEs. The significance level was set at *p* ≤ 0.05. The analyses were performed using the statistical software SPPS Statistics Version 24.

## 3. Results

From the cohort of 1000 RA patients, 355 patients were ≥60 years old and were screened for inclusion in the study. Of them, 133 patients were excluded because MTX was not prescribed during the follow-up, and 26 additional patients were excluded because they were prescribed biological therapy alongside MTX. Therefore, 198 elderly RA patients met the inclusion criteria and were included in the study.

Table 1 describes the sociodemographic and disease characteristics of the 198 elderly RA patients included in this study. Most of them were females (84.8%), with a mean age of 66.8 ± 5.6 years. The median RA disease duration was 1 year at the cohort onset, and 40.9% had a positive rheumatoid factor (RF). Of them, 65.7% had extraarticular manifestations, the three most frequent being anemia (35.4%), secondary Sjogren syndrome (34.8%) and rheumatoid nodules (23.7%).

### 3.1. Pharmacological Treatment

Table 2 describes the pharmacological treatment prescribed by the rheumatologist at baseline. Monotherapy with MTX at the beginning of their treatment was reported in 120 patients (60.6%), while 78 patients (39.3%) used combined therapy since the onset of treatment, the most frequent combination being MTX plus Sulfasalazine (SSZ) (19.2%) and MTX plus Chloroquine (CHL) (8.6%). Polypharmacy (≥5 drugs taken simultaneously by ≥90 days) including all the drugs taken by the patients at the baseline was observed in almost all the patients (94.9%). Folate supplementation was prescribed for 77.8% of the patients. The MTX dose is reported as the last dosage reported.

### 3.2. Changes of MTX Monotherapy to MTX Combined Therapy during the Follow-Up

The rate of using MTX as monotherapy decreased during the follow-up. After 5 years, 105 patients remained in the cohort; 44.8% patients used monotherapy with MTX and 55.2% combined therapy, the most frequent combinations being MTX plus SSZ (21.0%) and MTX plus Leflunomide (LEF) (9.5%). After 10 years, only 44 patients remained in the cohort; 29.5% patients used monotherapy with MTX and 70.5% combined therapy, the most used combination being MTX plus SSZ (22.7%). At 15 years, only 12 patients remained in the study; 30.6% were using MTX monotherapy and 55.1% combined therapy, with MTX plus SSZ (24.5%) as the most frequent combination of cs-DMARDs.

### 3.3. Adverse Events Leading to Suspension of MTX

Table 3 describes the adverse events that led to the suspension of MTX. During follow-up, 64/198 patients (32.3%) suspended MTX; of those, 54 (27.3%) withdrawals were due to adverse events, 5 (2.5%) were due to drug shortage and only 2 patients (1.0%) suspended MTX due to inefficacy. For the adverse events motivating definitive suspension of MTX, for 54 (27.3%), the main organs/systems involved were gastrointestinal (15.7%), mucocutaneous (3.0%), hepatic (2.5%) and constitutional symptoms (2.0%). In data that are not shown in tables, 16 patients (29.6%) had severe adverse events, such as: transaminasemia, bleeding diverticulitis and upper digestive tract bleeding, interstitial pneumopathy and/or pulmonary fibrosis, and severe infections.

### 3.4. Rate of MTX Withdrawal

**Figure 1:**
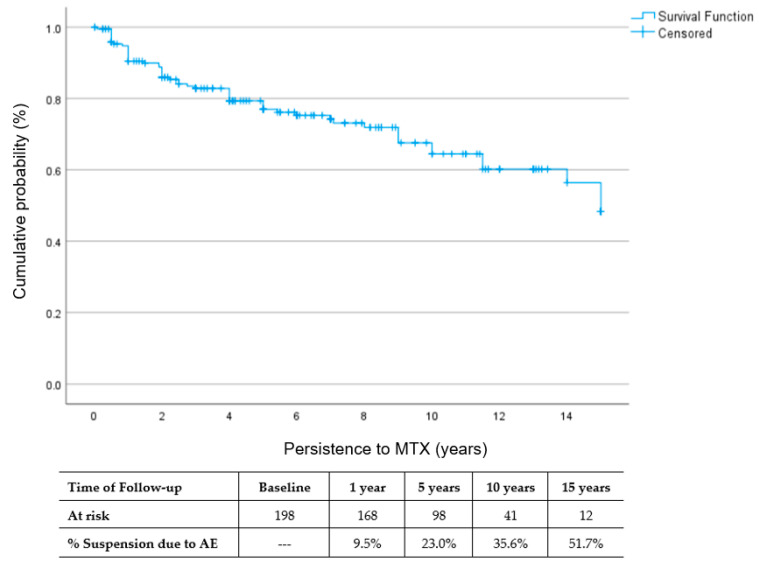
Persistence of MTX.

The patients using MTX were followed up for a total of 1155.20 person-years (mean: 6.1 ± 4.9 years, median: 5.0 years). Density incidence of MTX suspension caused by adverse events was 0.040 per 1000 person-years. Figure 1 shows Kaplan–Meier survival curves of the time to MTX withdrawal, in which the cumulative incidence of MTX suspension was 23.0% after 5 years, 35.6% after 10 years and 51.7% after 15 years.

Table 4 shows a comparison of variables observed at baseline between patients who discontinued MTX with those who continued MTX. There were no differences in epidemiological variables (such as: age, BMI and comorbidities), nor variables associated with RA (tender joint count, swollen joint count, morning stiffness or extraarticular manifestations) between these groups. Patients who discontinued MTX had higher doses of this DMARD compared to patients who did not discontinue that DMARD (*p* = 0.006). Instead, patients who did not discontinue MTX had higher frequency of using folic acid supplements compared to those who discontinued MTX (*p* < 0.001). Patients who continued MTX had a longer time of using this DMARD compared to those who withdrew MTX (*p* < 0.001).

**Figure 2:**
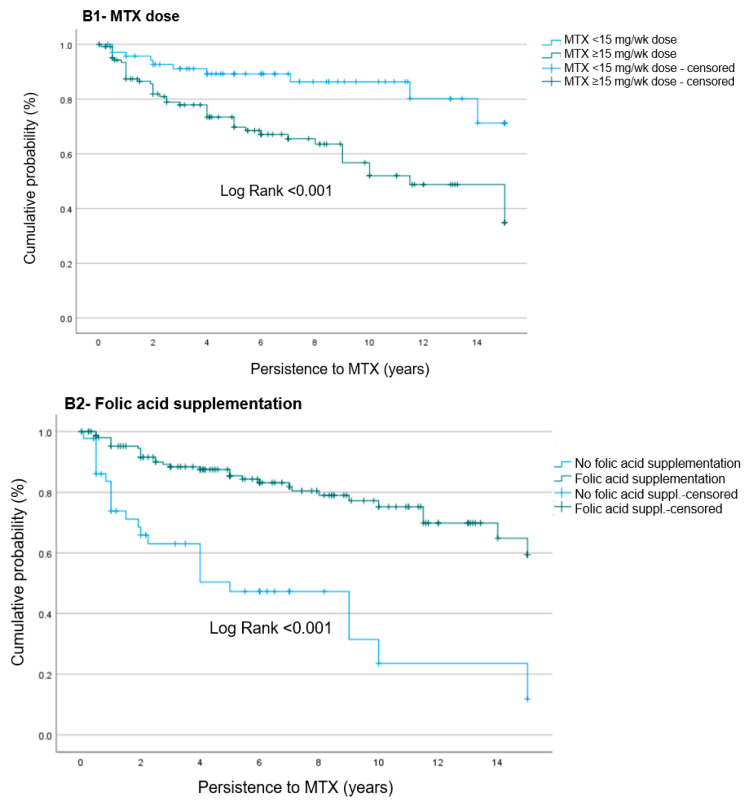

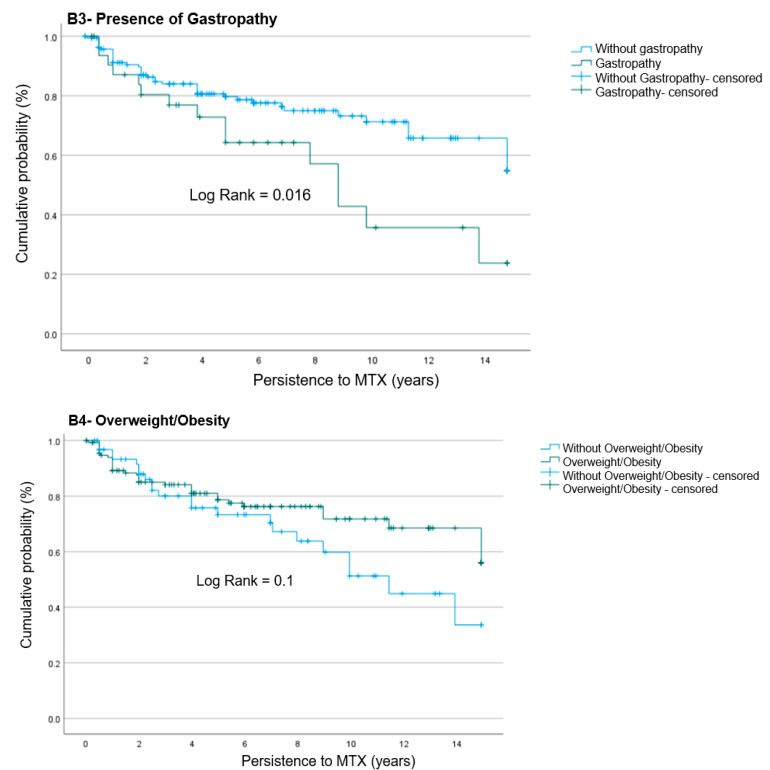
B1–B4: Risk factors of MTX discontinuation due to adverse events.

In Figure 2B1–B4, we showed different risk factors associated with MTX discontinuation by Kaplan–Meier analyses: Figure 2B1 describes the relation of suspension due to AEs with higher MTX doses (≥15 mg/wk). Patients with higher doses of MTX had significantly higher rate of suspension for MTX due to AEs (*p* < 0.001).

Figure 2B2 shows the comparison between survival on taking MTX in patients using folate supplementation vs. patients without it. Patients receiving folate supplementation had significantly lower rate of suspension for MTX due to AEs (*p* < 0.001). Figure 2B3 evaluates the effect of having gastropathy at baseline. Patients with gastropathy had significantly higher rate of suspension for MTX due to AEs (*p =* 0.016).

Other risk factors were evaluated, such as the number of DMARDs at the beginning of the treatment, the presence of comorbidities, anemia and BMI >25 (Overweight to obesity, Figure 2B4); though none of these reported an effect over MTX survival.

Table 5 shows the results of the multivariate Cox risk analysis. In the model, we included as time-dependent variable: MTX withdrawals due to adverse events. Covariables (potential confounders) tested in the unadjusted model (enter method) were female sex, overweight/obesity, presence of two or more comorbidities, tender joints count, swollen joints count, use of two or more DMARDs (MTX plus at least another DMARD), MTX dose equal to or higher than 15 mg/wk and folic acid supplementation. The risk model showed significant relations between MTX survival and the MTX dose ≥ 15 mg/wk (HR = 2.76, 95% CI: 1.33, 5.74, *p* = 0.006) and folic acid supplementation (HR = 0.27, 95% CI: 0.15, 0.49, *p* < 0.001), whereas no statistical associations were observed with the rest of covariables. In the second analysis, adjusting by stepwise method these potential confounders, in the model, only 2 variables remained significantly associated with risk of MTX withdrawals due to adverse events: MTX dose ≥ 15 mg/wk increasing the risk of MTX suspension (aHR = 2.46, 95% CI: 1.22, 4.96, *p* = 0.012) and folic acid supplementation as protective factor (aHR = 0.28, 95% CI: 0.16, 0.49, *p* < 0.001).

## 4. Discussion

This study identified that the incidence rates of definitive suspension of MTX due to AEs were 9.5% at 1 year, 23.0% at 5 years, 35.6% at 10 years and 51.7% at 15 years from initiating the drug. The main risk factor that increased the probability of MTX suspension due to AEs was the use of higher doses of MTX (≥15 mg/wk), whereas the use of folic acid acted as a protective factor against suspension due to AEs. The most frequent cause of MTX definitive suspension in RA elderly patients was adverse events, mainly gastrointestinal and mucocutaneus effects.

### 4.1. MTX Persistence

In the present study, we identified that 59.1% of elderly patients had MTX prescribed as a monotherapy at baseline and 29.7% in combined therapy. Monotherapy with MTX is more frequently indicated in elderly patients compared to young patients where the use of combined therapy with cs-DMARDS seems to be more frequently prescribed. In elderly RA patients, a concern is the increasing of side effects by pharmacological interactions of the high number of drugs used to treat comorbidities; therefore, it is more usual in our centers to prescribe MTX monotherapy in this population compared to younger RA patients. This therapeutic behavior has been reported by other studies: Tutuncu Z et al. compared in a cross-sectional design the information of two databases in different populations defined by age: the first was the information derived from the elderly-onset rheumatoid arthritis (EORA) database and the second derived from information of the younger-onset rheumatoid arthritis (YORA) database; these authors described a higher proportion of using MTX monotherapy in elderly RA compared to younger RA patients where the utilization of combined therapy was more usual (*p* = 0.005) [20]. Mathieu S et al. followed for five years French RA patients, stratified in three different age groups: >50, 50 to 64, and 65 to 74 years old, observing a higher prevalence (68.4%) of MTX monotherapy in their elderly group (65 to 74 years old), observing higher rates of MTX suspension correlated with the increase of other cs-DMARDS co-utilization, although the oldest group showed a higher persistence to RA treatment regardless of the number of drugs received [21]. Similarly, in our study, we did not identify statistical differences in the rate of suspension of MTX by AEs when it was used in combined therapy compared against use of MTX monotherapy.

We identified a low rate (9.5%) of MTX suspension due to AEs at 1 year of treatment; however, it increased to 23.0% at 5 years, and continued increasing to almost one of three patients (35.6%) at 10 years, and lastly, around one of each two patients (51.7%) suspended treatment at 15 years. Two studies performed in Danish and French RA patients identified a similar rate of MTX suspension at 5 years of treatment onset [15,21]. Bliddal H. et al. found a rate of MTX suspension in Danish RA patients (mean age 59.8 ± 14.4 years) similar to the one observed by us, of 25% at 5 years [15]. Mathieu S et al. observed in French RA patients, stratified in three groups as stated above, the rate of MTX suspension of 25% at 5 years, showing a higher retention rate in the oldest group [21]. Other authors have reported higher rates of MTX suspensions in their cohorts. Alarcon G.S. et al. reported a 55% rate of suspending MTX at 5 years due to toxicity in North American RA patients (mean age: 61.2 ± 12.4 years) [9]. Scully C.J. et al., in North American RA patients (mean age: 51 ± 12 years), observed that almost four of five patients (71.5%) suspended MTX at 5 years [22]. Lastly, Ideguchi H et al. reported that in Japanese RA patients (mean age 57.6 ± 11.4 years), there was a 40% rate of MTX suspension at 5 years [14]. Rate of MTX suspension due to AEs at 10 years was 35.6%, similar to those reported in Danish patients by Bliddal H, observing that at 10 years, 35% of patients stopped MTX treatment [15]. Other cohorts reported higher rates of suspension at 10 years. Hoesktra M et al. reported that in Dutch RA patients (median age: 59.7), at 9 years, the rate of MTX withdrawal was 50% [10]; Alarcon G.S. et al. observed a higher discontinuation rate of 70% in RA patients after 10 years (mean age: 61.2 ± 12.4) [8]. Lastly, to the best of our knowledge, we could not identify a cohort with a follow-up of 15 years of elderly patients, where one of each two patients (51.7%) had suspended treatment at the end of the study period.

### 4.2. Adverse Events of MTX That Led to Suspension

The main EAs reported in our study were gastrointestinal (15.7%), mucocutaneus (3.0%) and hepatic (2.5%). We identified several studies in which Aes were associated with the main cause of MTX suspension. Alarcon G.S. et al. described that the most frequent AEs leading to discontinuation of MTX in North American RA patients (mean age: 61.2 ± 12.4 years) were gastrointestinal events (43.8%) followed by AEs related to skin involvements (22.8%) [9]. Nikiphorou E et al. reported in a cohort of UK RA patients (mean age of the patients was not specified) that side effects were the main reason for MTX withdrawal, of which, gastrointestinal events were the most frequent adverse effects (32.7%) [23]. In a more recent study, Nagafuchi H et al. reported in a retrospective cohort performed in Egyptian RA patients (with median age: 58.0 years) that the most frequent causes of withdrawals were infections (20.0%), malignancies (14.1%) and respiratory disorders (10.2%) [24]. In comparison, the rate of infections and respiratory disorders as causes for suspension were significantly lower (5.4% and 3.7%, respectively), and no malignancies were reported in our cohort. Instead, the main cause of suspension was gastrointestinal effects like the causes reported in cohorts from North American patients [9]. Other cohort studies have reported the incidence of AEs in RA patients treated with DMARDs. Singal V. et al. analyzed the AEs in young patients from India (mean age: 38 years) observing abnormal level of transaminases (29.1%) and excessive nausea along with vomiting (16.6%); however, this cohort did not focus on treatment suspension [25]. Sherbini A. A. et al., in a prospective cohort, analyzed the rates and baseline predictor of AEs observed in the first year of MTX treatment in RA patients from the UK (mean age: 59 years); at twelve months, 77.5% of their patients reported at least one adverse event, mainly gastrointestinal (42.0%), constitutional (39.6%), neurological (28.6%), mucocutaneus (26.0%) and pulmonary (20.9%). Nevertheless, they did not extend the time of follow-up in this interesting cohort [26]. Takahashi C et al. reported in a 76 week study, 34% of 79 Japanese patients with RA (mean age: 56.7) had AEs, mainly gastrointestinal symptoms and hepatotoxicity [27]. Cummins L et al. described in a prospective cohort of 181 Australian RA patients (mean age: 52 years) who received a combination of triple DMARDs including MTX + SSZ + HCQ, the tolerability, persistence and efficacy. Of them, MTX was withdrawn in 29% of their patients, in this cohort, the gastrointestinal intolerance (15.0%) and rash (11.0%) being the most frequent AEs [28]. All these results reflect the importance of surveilling gastrointestinal and cutaneous AEs in users of MTX and support our study findings.

### 4.3. Risk Factors Associated with MTX Suspension Due to AEs

We identified the risk factors associated with the suspension of MTX therapy due to adverse events. A MTX dose ≥ 15 mg/wk was found to increase 2.46-fold the risk of the suspension of MTX due to AEs. Two studies analyzed RA patients in which MTX withdrawal was associated with the use of lower doses of MTX compared to the doses observed in our patients. Ideguchi H et al., in Japanese RA patients (mean age: 57.6 ± 11.4 years), reported in their cohort study that MTX discontinuation is associated with lower MTX doses (>8 mg/wk), increasing the risk up to almost 3-fold [14]. Asai S et al., in a cross-sectional study in Japanese patients (Median age: 64), observed that MTX doses >8 mg/wk increased the risk of reflux and abdominal pain (OR: 1.62 and 1.62, respectively); however, association between these AEs and MTX suspension was not analyzed [29]. Shoda H. et al. performed a cohort in Japanese RA patients (mean age: 60.8), comparing the maintenance dose of MTX in patients who had AEs vs. RA patients without AEs, identifying a relation between higher doses and AEs (9.6 ± 4.2 mg/wk vs. 6.7 ± 3.0 mg/wk respectively, *p* = 0.03) [30]. These data support our results of a higher risk of withdrawals of this DMARD related to higher doses.

### 4.4. Folate Supplementation as Protective Factor for MTX Suspension Due to AEs

In our study, folic acid supplementation was observed as a protective factor associated with MTX persistence, reducing the probability of MTX suspension due to AEs by 0.28-fold. Two other studies also identified the supplementation of folic acid as a protective factor in MTX therapy. Ideguchi H et al., in Japanese RA patients (mean age: 57.6 ± 11.4 years), reported that a lack of folic acid supplementation increases the risk of MTX discontinuation (RR = 1.93, *p* = 0.029), although it is not directly associated with AE development [14]. Hoekstra M et al., in their cohort of Dutch RA patients (median age: 59.7), reported that folate supplementation was associated with MTX survival, acting as a protective factor (RR = 0.25, *p* < 0.001) [10].

### 4.5. Strengths and Limitations

The present study focused on MTX suspension due to AEs in elderly RA patients. There is limited information regarding suspension of MTX due to AEs in elderly RA patients. Our study shows that 19% to 35% of patients in our cohort of RA patients are aged ≥ 60 years old. Elderly RA patients represent a challenge for clinicians treating this subgroup of the population because they have an impairment in metabolism and excretion of several drugs, making them more susceptible to suffer adverse events linked to the drugs used for their treatments. The present study described the long-term incidence of suspension of MTX due to AEs. We identified that at 10 years, the rate of patients that suspended MTX was 35.6%, and at 15 years this increased up to 51.7%. The main AEs identified were gastrointestinal and mucocutaneous. Risk predictors were analyzed after using an adjusted multivariate analysis, the main risk factor being a higher dose of MTX and as a protective factor, the utilization of folic acid.

However, this study has several limitations; the main one of them is derived from the retrospective cohorts where we cannot exclude the possibility that some information regarding several other risk factors could be missing. Among them, we have no information regarding genetic variables, MTX and its metabolites serum levels, therapeutic adherence or other variables that may influence the development of AEs but are not usually registered in the clinical charts. Another limitation observed in long-term retrospective cohorts is the loss of patients during the follow-up; in this cohort, the number of patients who were censored during the study was increased by other causes different to AEs. We therefore used the density of incidence as the strategy to identify the rate of therapeutic suspension of MTX, whereas prospective cohort studies with lower follow-up could use cumulative incidence as a better measure of incidence. We adjusted the time for developing events using Kaplan–Meir curves and hazard ratio in the multivariable Cox regression analysis. One potential limitation in our study was the use of 1987 ACR criteria for the inclusion of patients with a diagnosis of RA; although these criteria have a good sensitivity (79%) and specificity (90%) in patients with established RA, these values can decrease in patients with early RA (sensitivity of 77% and specificity 77%) [31]. However, at the time of the cohort onset, this set of criteria were the most used in the clinical settings in our country. Another additional limitation of this cohort was the lack of data regarding the positivity of anti-CCP antibodies; these antibodies have been related to several outcomes including erosions [32] and some extraarticular manifestations (such as subcutaneous nodules and lung involvement) [33]. In this cohort, an assessment of anti-CCP antibodies at the onset and their relation with adverse events of MTX was not investigated. Finally, we excluded elderly RA patients treated with biologic DMARDs; although, nowadays, the use of biologic therapy is more prescribed in our institution, at the time of the cohort onset, only 10% of the patients had this type of therapy.

## 5. Conclusions

In elderly RA patients, the suspension of MTX usually occurs earlier in therapy, while as many as half of the patients tolerate it up to 15 years. The most frequent reason for suspending MTX is adverse events of a gastrointestinal and mucocutaneus nature. Higher doses of MTX were associated with an increased risk of suspension, while folate supplementation considerably improved MTX survival.

## Figures and Tables

**Table 1:** Characteristics of patients with rheumatoid arthritis.

	*n* = 198 (100.0)
Female gender, n (%)	168 (84.8)
Age (yrs), mean ± SD	66.8 ± 5.6
BMI *, mean ± SD	27.5 ± 5.7
Smoking, n (%)	6 (3.0)
Alcohol abuse, n (%)	4 (2.0)
Comorbidities, n (%)	153 (77.3)
Number of comorbidities, median (range)	1 (0.0, 4.0)
Overweight or Obesity, n (%)	118 (59.6)
Arterial Hypertension, n (%)	91 (46.0)
Osteoporosis, n (%)	57 (28.8)
Type 2 Diabetes Mellitus, n (%)	35 (17.1)
Clinical depression, n (%)	19 (9.6)
RA duration (yrs) up to onset of MTX treatment, median (range)	1.0 (0.0, 39.0)
Steinbroker’s functional class, n (%)	
- Functional class I, n (%)	49 (24.7)
- Functional class II–IV, n (%)	149 (75.3)
Pain score (VAS ** 0–100), mean ± DS	72.5 ± 15.7
Morning stiffness (>1 h), n (%)	108 (54.5)
Positive RF ***, n (%)	81 (40.9)
Extraarticular manifestations, n (%):	130 (65.7)
Anemia, n (%)	70 (35.4)
Sjogren syndrome, n (%)	69 (34.8)
Rheumatic nodules, n (%)	47 (23.7)
Neuropathies, n (%)	39 (19.7)
Pneumopathies, n (%)	4 (2.0)

Abbreviations: * BMI: Body mass index, ** VAS: Visual analogue scale, *** RF: Rheumatoid factor. Qualitative variables are expressed as frequencies and percentages, and quantitative variables as means and standard deviations (SDs) or as medians with minimum and maximum values (range).

**Table 2:** Baseline pharmacological treatment.

	*n* = 198 (100.0)
Methotrexate as monotherapy, n (%)	120 (60.6)
Combined therapy, n (%)	78 (39.3)
MTX dose (mg)/wk, mean ± SD	13.6 ± 3.6
Usage duration (yrs), median (min., max.)	5 (0.01, 15.00)
Combined therapy MTX plus:	
Sulfasalazine, n (%)	38 (19.2)
Azathioprine, n (%)	17 (8.6)
Leflunomide, n (%)	9 (4.5)
Chloroquine, n (%)	4 (2.0)
SSZ + CHQ, n (%)	5 (2.5)
AZA + LEF, n (%)	2 (1.0)
Other combinations *, n (%)	3 (1.5)
Glucocorticoids, n (%)	169 (85.4)
NSAIDs, n (%)	189 (95.5)
Analgesics, n (%)	156 (78.8)
Other drugs:	
Omeprazole, n (%)	130 (65.7)
Antiresorptive, n (%)	46 (23.2)
Antihypertensives, n (%)	43 (21.7)
Antidiabetic drugs, n (%)	16 (8.1)
Num. of drugs used at the same time, mean ± SD	8.3 ± 2.6
Polypharmacy (≥5 or more drugs), n (%)	188 (94.9)
Folic acid supplementation, n (%)	154 (77.8)

Abbreviatures: MTX: Methotrexate, SSZ: Sulfasalazine, CHQ: Chloroquine, AZA: Azathioprine, LEF: Leflunomide, NSAIDs: Non-steroidal anti-inflammatory drug. Qualitative variables are expressed as frequencies and percentages, and quantitative variables as medians (ranges) or means and standard deviations (SDs). * Other combinations: MTX + SSZ + CHQ (0.5%), MTX + SSZ + LEF (0.5%) and MTX + CHQ + LEF (0.5%).

**Table 3:** Classification of adverse events reported that led to discontinuation.

Patients Who Discontinued MTX Due to AEs	*n* = 54 (100.0)
Main organs and systems with AEs	
Gastrointestinal, n (%)	31 (57.4)
Mucocutaneous, n (%)	6 (11.1)
Hepatic, n (%)	5 (9.2)
Constitutional symptoms, n (%)	4 (7.4)
Recurrent Infections, n (%)	3 (5.5)
Hematologic, n (%)	2 (3.7)
Pulmonary, n (%)	2 (3.7)
Renal, n (%)	1 (1.8)
Specific adverse event	
Epigastralgia and/or Gastritis	18 (33.3)
Nausea, vomiting, gastric intolerance and/or diarrhea	10 (18.5)
Transaminitis	5 (9.2)
Bleeding diverticulitis and upper digestive tract bleeding	5 (9.2)
Oral ulcers and/or Alopecia/hair loss	5 (9.2)
Leukopenia and/or lymphopenia	2 (3.7)
Interstitial pneumopathy/pulmonary fibrosis	2 (3.7)
Weakness and weight loss	2 (3.7)
Dermatosis	1 (1.8)
Tuberculosis infection.	1 (1.8)
Hepatitis C infection	1 (1.8)
Recurrent infections	1 (1.8)
Urinary lithiasis	1 (1.8)

Qualitative variables are expressed as frequencies and percentages, and quantitative variables as medians (ranges) or means and standard deviations (SDs).

**Table 4:** Comparison of variables between RA patients with MTX withdrawals due to adverse events (AEs) vs. RA patients who continued MTX.

Variable, n (%)	MTX Withdrawals * *n* = 54 (100.0)	MTX (Non-Withdrawals) *n* = 144 (100.0)	*p*
Age (yrs), mean ± SD	65.6 ± 5.0	67.2 ± 5.7	0.08
BMI, mean ± SD	28.2 ± 7.5	27.2 ± 4.5	0.3
Num. comorbidities, mean ± SD	1.2 ± 1.1	1.3 ± 0.9	0.6
Arterial Hypertension, n (%)	25 (46.3)	66 (45.8)	0.9
RA duration before MTX, mean ± DS	5.6 ± 8.7	5.3 ± 7.9	0.8
Pain score (VAS 0–100 mm), mean ± DS	74.2 ± 14.6	73.3 ± 14.0	0.7
Tender joints count, mean ± DS	10.3 ± 5.9	10.4 ± 6.0	0.8
Swollen joints count, mean ± DS	9.9 ± 4.4	9.3 ± 4.1	0.4
Morning stiffness (>1 h), n (%)	30 (55.6)	78 (54.2)	0.8
Extraarticular manifestations, n (%):	40 (74.1)	90 (62.5)	0.1
Sjogren syndrome, n (%)	14 (9.7)	7 (13.0)	0.5
Rheumatic nodules, n (%)	13 (24.1)	34 (23.6)	0.9
RF, n (%)	23 (42.6)	58 (40.3)	0.7
MTX dose (mg/wk), mean ± SD	14.3 ± 3.1	12.9 ± 3.2	0.006
Time of using MTX (years), mean ± SD	4.1 ± 4.0	6.4 ± 4.4	<0.001
Combined therapy **, n (%)	21 (38.9)	57 (39.6)	0.9
Glucocorticoids, n (%)	50 (92.6))	119 (82.6)	0.07
Polypharmacy (≥5 or more drugs), n (%)	50 (92.6)	138 (95.8)	0.3
Folic acid supplementation, n (%)	30 (55.6)	124 (86.1)	<0.001

Abbreviatures: RA: Rheumatoid Arthritis, MTX: Methotrexate, BMI: Body Mass Index, VAS: Visual Analogue Scale, RF: Rheumatoid Factor * MTX withdrawals due to AEs. ** Combined therapy was considered as patients with MTX + at least 1 or more DMARDs. All variables were reported at cohort onset excepting: (1) MTX dose: this variable was reported as the last dose before suspending the drug (in patients with MTX suspension), or as the last MTX dose registered in the clinical chart in those patients who did not discontinue this drug; (2) Time of using MTX, this variable was reported computing the years of using that DMARD until drug suspension; in those who continued MTX this variable was computed as the total time since MTX onset until the last visit. Comparisons between proportions were performed using chi-square test and comparisons between means were performed using Student *t*-tests.

**Table 5:** Risk factors for MTX suspension due to adverse events in elderly patients with rheumatoid arthritis.

	MTX Treatment Suspension Due to Adverse Events					
	Unadjusted			Adjusted		
	Enter Method			Stepwise Method		
	HR	95% CI	*p*-Value	aHR	95% CI	*p*-Value
Female sex	1.03	0.47–2.28	0.9	--	--	--
Overweight/obesity	0.75	0.41–1.36	0.3	--	--	--
≥2 Comorbidities	0.70	0.33–1.46	0.3	--	--	--
Tender joints	0.98	0.93–1.03	0.6	--	--	--
Swollen joints	1.05	0.98–1.13	0.1	--	--	--
≥2 DMARDs *	0.78	0.43–1.41	0.4	--	--	--
MTX dose ≥ 15 mg/wk	2.76	1.33–5.74	0.006	2.46	1.22–4.96	0.012
Folic acid usage	0.27	0.15–0.49	<0.001	0.28	0.16–0.49	<0.001

Abbreviations: BMI: Body mass index. DMARDs: Disease-modifying antirheumatic drugs. MTX: Methotrexate. * For covariates the use of MTX plus one or more DMARDS (SSZ, CLQ, AZA and/or LEF) was considered as ≥2 DMARDs. aHR: Adjusted hazard ratio. 95 CI: 95% Confidence interval. Crude HRs were obtained using the enter method. aHR was obtained using the stepwise method. Variables excluded from the final model were female sex, ≥25 BMI, ≥2 comorbidities, tender joints, swollen joints and ≥2 DMARDs.

## Data Availability

The dataset supporting the conclusions presented in this article is available on request from the corresponding author on reasonable request.

## References

[1] Smolen J.S., Aletaha D., Barton A., Burmester G.R., Emery P., Firestein G.S., Kavanaugh A., McInnes I.B., Solomon D.H., Strand V. (2018). Rheumatoid Arthritis. Nat. Rev. Dis. Primers.

[2] Silman A.J., Pearson J.E. (2002). Epidemiology and genetics of rheumatoid arthritis. Arthritis Res..

[3] NICE (2018). Rheumatoid Arthritis in Adults: Management. www.nice.org.uk/guidance/ng100.

[4] Smolen J.S., Landewé R.B.M., Bergstra S.A., Kerschbaumer A., Sepriano A., Aletaha D., Caporali R., Edwards C.J., Hyrich K.L., Pope J.E. (2023). EULAR recommendations for the management of rheumatoid arthritis with synthetic and biological disease-modifying antirheumatic drugs: 2022 update. Ann. Rheum. Dis..

[5] Zhao Z., Hua Z., Luo X., Li Y., Yu L., Li M., Lu C., Zhao T., Liu Y. (2022). Application and pharmacological mechanism of methotrexate in rheumatoid arthritis. Biomed. Pharmacother..

[6] Goycochea-Robles M.V., Arce-Salinas C.A., Guzmán-Vázquez S., Cardiel-Ríos M.H. (2007). Prescription Rheumatology Practices Among Mexican Specialists. Arch. Med Res..

[7] Moura C.S., Schieir O., Valois M., Thorne C., Bartlett S.J., Pope J.E., Hitchon C.A., Boire G., Haraoui B., Hazlewood G.S. (2020). Treatment Strategies in Early Rheumatoid Arthritis Methotrexate Management: Results from a Prospective Cohort. Arthritis Care Res..

[8] Alarcon G.S., Tracy I.C., Strand G.M., Singh K., Macaluso M. (1995). Survival and drug discontinuation analyses in a large cohort of methotrexate treated rheumatoid arthritis patients. Ann. Rheum. Dis..

[9] Alarcóan G.S., Tracy I.C., Blackburn W.D. (1989). Methotrexate in rheumatoid arthritis. Toxic effects as the major factor in limiting long-term treatment. Arthritis Rheum..

[10] Hoekstra M., Laar M.A.F.J.V.D., Moens H.J.B., Kruijsen M.W.M., Haagsma C.J. (2003). Longterm observational study of methotrexate use in a Dutch cohort of 1022 patients with rheumatoid arthritis. J. Rheumatol..

[11] Sevillano Gutierrez J., Capelusnik D., Schneeberger E., Citera G. (2019). Tolerancia, sobrevida y adherencia al tratamiento con Metotrexato en pacientes con artritis reumatoidea. Rev. Argent. De Reumatol..

[12] Sherbini A.A., Sharma S.D., Gwinnutt J.M., Hyrich K.L., Verstappen S.M.M. (2021). Prevalence and predictors of adverse events with methotrexate mono- and combination-therapy for rheumatoid arthritis: A systematic review. Rheumatology.

[13] Verstappen S.M.M., Bakker M.F., Heurkens A.H.M., van der Veen M.J., A Kruize A., Geurts M.A.W., Bijlsma J.W.J., Jacobs J.W.G. (2010). Adverse events and factors associated with toxicity in patients with early rheumatoid arthritis treated with methotrexate tight control therapy: The CAMERA study. Ann. Rheum. Dis..

[14] Ideguchi H., Ohno S., Ishigatsubo Y. (2007). Risk Factors Associated with the Cumulative Survival of Low-Dose Methotrexate in 273 Japanese Patients with Rheumatoid Arthritis. J. Clin. Rheumat..

[15] Bliddal H., Eriksen S.A., Christensen R., Lorenzen T., Hansen M.S., Østergaard M., Dreyer L., Luta G., Vestergaard P. (2015). Adherence to Methotrexate in Rheumatoid Arthritis: A Danish Nationwide Cohort Study. Arthritis.

[16] World Health Organization Ageing and Health. https://www.who.int/news-room/fact-sheets/detail/ageing-and-health#:~:text=By%202050%2C%20the%20world's%20population,2050%20to%20reach%20426%20million.

[17] Morales-Romero J., Cázares-Méndez J.M., Gámez-Nava J.I., Triano-Páez M., Villa-Manzano A., López-Olivo M., Rodríguez-Arreola B., González-López L. (2005). La atención médica en reumatología en un hospital de segundo nivel de atención [Patterns of health care in an out patient rheumatologic clinic]. Reumatol. Clin..

[18] Arnett F.C., Edworthy S.M., Bloch D.A., McShane D.J., Fries J.F., Cooper N.S., Healey L.A., Kaplan S.R., Liang M.H., Luthra H.S. (1988). The American Rheumatism Association 1987 revised criteria for the classification of rheumatoid arthritis. Arthritis. Rheum..

[19] World Medical Association (2013). World Medical Association Declaration of Helsinki: Ethical principles for medical research involving human subjects. JAMA.

[20] Tutuncu Z., Reed G., Kremer J., Kavanaugh A. (2006). Do patients with older-onset rheumatoid arthritis receive less aggressive treatment?. Ann. Rheum. Dis..

[21] Mathieu S., Pereira B., Saraux A., Richez C., Combe B., Soubrier M. (2021). Disease-modifying drug retention rate according to patient age in patients with early rheumatoid arthritis: Analysis of the ESPOIR cohort. Rheumatol. Int..

[22] Scully C.J., Anderson C.J., Cannon G.W. (1991). Long-term methotrexate therapy for rheumatoid arthritis. Semin. Arthritis Rheum..

[23] Nikiphorou E., Negoescu A., Fitzpatrick J.D., Goudie C.T., Badcock A., Östör A.J.K., Malaviya A.P. (2014). Indispensable or intolerable? Methotrexate in patients with rheumatoid and psoriatic arthritis: A retrospective review of discontinuation rates from a large UK cohort. Clin. Rheumatol..

[24] Nagafuchi H., Goto Y., Kiyokawa T., Kawahata K. (2022). Reasons for discontinuation of methotrexate in the treatment of rheumatoid arthritis and challenges of methotrexate resumption: A single-center, retrospective study. Egypt. Rheumatol. Rehabil..

[25] Singal V., Chaturvedi V., Brar K. (2005). Efficacy and Toxicity Profile of Methotrexate Chloroquine Combination in Treatment of Active Rheumatoid Arthritis. Med. J. Armed Forces India.

[26] Sherbini A.A., Gwinnutt J.M., Hyrich K.L., Verstappen S.M.M., Adebajo A., Ahmed K., Al-Ansari A., Amarasena R., Bukhari M., RAMS Co-Investigators (2022). Rates and predictors of methotrexate-related adverse events in patients with early rheumatoid arthritis: Results from a nationwide UK study. Rheumatology.

[27] Takahashi C., Kaneko Y., Okano Y., Taguchi H., Oshima H., Izumi K., Yamaoka K., Takeuchi T. (2017). Association of erythrocyte methotrexate-polyglutamate levels with the efficacy and hepatotoxicity of methotrexate in patients with rheumatoid arthritis: A 76-week prospective study. RMD Open.

[28] Cummins L., Katikireddi V.S., Shankaranarayana S., Su K.Y.C., Duggan E., Videm V., Pahau H., Thomas R. (2015). Safety and retention of combination triple disease-modifying anti-rheumatic drugs in new-onset rheumatoid arthritis. Intern. Med. J..

[29] Asai S., Nagai K., Takahashi N., Watanabe T., Matsumoto T., Asai N., Sobue Y., Ishiguro N., Kojima T. (2019). Influence of methotrexate on gastrointestinal symptoms in patients with rheumatoid arthritis. Int. J. Rheum. Dis..

[30] Shoda H., Inokuma S., Yajima N., Tanaka Y., Oobayashi T., Setoguchi K. (2007). Higher maximal serum concentration of methotrexate predicts the incidence of adverse reactions in Japanese rheumatoid arthritis patients. Mod. Rheumatol..

[31] Banal F., Dougados M., Combescure C., Gossec L. (2009). Sensitivity and specificity of the American College of Rheumatology 1987 criteria for the diagnosis of rheumatoid arthritis according to disease duration: A systematic literature review and meta-analysis. Ann. Rheum. Dis..

[32] Sulaiman F.N., Wong K.K., Ahmad W.A.W., Ghazali W.S.W. (2019). Anti-cyclic citrullinated peptide antibody is highly associated with rheumatoid factor and radiological defects in rheumatoid arthritis patients. Medicine.

[33] Korkmaz C., Us T., Kaşifoğlu T., Akgün Y. (2006). Anti-cyclic citrullinated peptide (CCP) antibodies in patients with long-standing rheumatoid arthritis and their relationship with extra-articular manifestations. Clin. Biochem..

